# A 14.7 kDa Protein from *Francisella tularensis subsp. novicida* (Named FTN_1133), Involved in the Response to Oxidative Stress Induced by Organic Peroxides, Is Not Endowed with Thiol-Dependent Peroxidase Activity

**DOI:** 10.1371/journal.pone.0099492

**Published:** 2014-06-24

**Authors:** Diogo de Abreu Meireles, Thiago Geronimo Pires Alegria, Simone Vidigal Alves, Carla Rani Rocha Arantes, Luis Eduardo Soares Netto

**Affiliations:** Departamento de Genética e Biologia Evolutiva, Instituto de Biociências, Universidade de São Paulo, São Paulo, Brazil; University of Louisville, United States of America

## Abstract

*Francisella* genus comprises Gram-negative facultative intracellular bacteria that are among the most infectious human pathogens. A protein of 14.7 KDa named as FTN_1133 was previously described as a novel hydroperoxide resistance protein in *F. tularensis subsp. novicida*, implicated in organic peroxide detoxification and virulence. Here, we describe a structural and biochemical characterization of FTN_1133. Contrary to previous assumptions, multiple amino acid sequence alignment analyses revealed that FTN_1133 does not share significant similarity with proteins of the Ohr/OsmC family or any other Cys-based, thiol dependent peroxidase, including conserved motifs around reactive cysteine residues. Circular dichroism analyses were consistent with the *in silico* prediction of an all-α-helix secondary structure. The pK_a_ of its single cysteine residue, determined by a monobromobimane alkylation method, was shown to be 8.0±0.1, value that is elevated when compared with other Cys-based peroxidases, such as peroxiredoxins and Ohr/OsmC proteins. Attempts to determine a thiol peroxidase activity for FTN_1133 failed, using both dithiols (DTT, thioredoxin and lipoamide) and monothiols (glutathione or 2-mercaptoethanol) as reducing agents. Heterologous expression of *FTN_1133* gene in *ahpC* and *oxyR* mutants of *E. coli* showed no complementation. Furthermore, analysis of *FTN_1133* protein by non-reducing SDS-PAGE showed that an inter-molecular disulfide bond (not detected in Ohr proteins) can be generated under hydroperoxide treatment, but the observed rates were not comparable to those observed for other thiol-dependent peroxidases. All the biochemical and structural data taken together indicated that FTN_1133 displayed distinct characteristics from other thiol dependent peroxidases and, therefore, suggested that FTN_1133 is not directly involved in hydroperoxide detoxification.

## Introduction

*Francisella* genus, a group of Gram-negative facultative intracellular bacteria, comprises species that are among the most infectious human pathogens. Indeed, *F. tularensis subsp. tularensis* can infect human airways as few as 10 c.f.u., and if untreated, generally provokes a fatal outcome [1]. The other subspecies, *F. tularensis subsp. novicida*, *holarctica* and *mediasiatica*, in addition to other species, *F. philomiragia*, display reduced virulence to humans and rarely result in fatalities, acting like opportunistic pathogens [2], [3].

*F. tularensis* can infect many host cell types that include epithelial, endothelial, polymorphonuclear neutrophils and mononuclear phagocytes [4] and, although the exact mechanism of the course of infection is under active research, it is well established that *F. tularensis* is able to prevent the oxidative burst by inhibition of NADPH oxidase complex (NOX2) activity, the main Reactive Oxygen Species (ROS) generation machinery of the phagocytic cell [1]. Besides NADPH oxidases, phagocytic cells are also house of other oxidative systems such as nitric oxide synthases and heme-peroxidases that also play decisive role in microbial clearance [5]–[8].

Although *F. tularensis* seems to preferentially utilize mechanisms dedicated to inhibit ROS generation by the NADPH oxidase complex, some proteins directly involved in ROS decomposition are also recruited during the infectious process [9], probably protecting this pathogen from oxidative insults and interfering with macrophage signaling and cytokine production [10]. Indeed, analysis of *F. tularensis* genome revealed the occurrence of genes that are directly involved in ROS detoxification. For example, in the genome of *Francisella tularensis subsp. novicida* U112, it is observed the presence of *sodB* and *sodC* genes (for Fe and Cu/Zn superoxide dismutases, respectively); *katG*, which encodes a bifunctional catalase/peroxidase enzyme; *FTN_0698*, which encodes a glutathione peroxidase (GPx); and *FTN_1624*, a member of DyP-type peroxidase family [11]. Furthermore, genes encoding three putative peroxiredoxin enzymes were found: FTN_0973, which belongs to the AhpC/Prx1 sub-family; FTN_0958, a putative Prx5 sub-family member; and FTN_1756, a BCP (Bacterioferritin Comigratory Protein) protein that belongs to a BCP/PrxQ sub-family, according to a classification proposed by [12]. Remarkably, *Francisella* mutants of *sodBC* and *katG genes* have been shown to present reduced virulence [13]–[15].

FTN_1133 was recently identified in a macrophage replication screen as a protein involved in virulence and also as a novel hydroperoxide resistance protein in *F. tularensis subsp. novicida* [16]. FTN_1133 was proposed to be related to the Ohr/OsmC family of proteins due to the reduced capability of a FTN_1133 mutant strain to detoxify organic hydroperoxide and by sequence similarity to OhrA from *Bacillus megaterium*.

Ohr (Organic Hydroperoxide Resistance protein) belongs to a large family of proteins called Ohr/OsmC, which is subdivided into three sub-families: Ohr, OsmC and YhfA [17], [18]. Ohr enzymes are clearly implicated in the response of bacteria to the stress induced by organic hydroperoxides, whereas OsmC proteins are involved in the response to osmotic stress [18].With exception of YhfA subfamily, Ohr and OsmC are thiol-dependent peroxidase that converts organic hydroperoxides into their corresponding alcohols [19], [20]. Almost all Ohr/OsmC family members have two cysteine residues located in conserved motifs that represent a signature for this family. The catalytic mechanism involves reversible oxidation of a peroxidatic cysteine, located at N-terminus, to sulfenic acid (-SOH), by organic hydroperoxide, followed by formation of an intra-molecular disulfide bond with a resolution cysteine, located at C-terminus of protein. Finally, Ohr is reduced back to the sulfhydryl form by lipoylated proteins through electrons originated from NADH [21].

Here, we characterized FTN_1133 structurally and biochemically, in attempt to gain more evidences about its function as a putative thiol peroxidase. We observed that FTN_1133 displayed very distinct properties in comparison with proteins belonging to the Ohr/OsmC family and other thiol dependent peroxidases. Therefore, our results suggest that FTN_1133 does not present a thiol dependent activity and possibly possesses a distinct mechanism to detoxify organic hydroperoxides.

## Materials and Methods

### Strains and growth conditions

*E. coli* strains were growth in Luria Bertani (LB) medium at 37°C with appropriated antibiotics: ampicilin (100 µg/mL) or kanamycin (15 µg/mL).

### Cloning procedures

*FTN_1133* gene was commercially synthesized by GenScript USA Inc. containing the sites for *Nhe*I and *Xho*I restriction enzymes in the flanking regions. To clone into pET15b expression vector (Novagen), *FTN_1133* sequence was amplified by PCR, from pUC57 containing *FTN_1133* sequence, using the following primers 5′-CGATC**CATATG**GCTATTAGTCAAAATGTTATAAAAATAC-3′ and 5′-CGC**GGATCC**TTAGCTTTTATTATCGATCAAGCTTCG-3′, which contained sites for *Nde*I and *Bam*HI restriction enzymes (bold letters). *FTN_1133* gene was then sequenced using T7 promoter and terminator oligonucleotides. Cloning, expression, and purification of recombinant OsmC, from *E. coli* and Ohr, from *Xyllela fastidiosa*, were performed as described previously in [21] and [19], respectively.

### Bioinformatics analysis

Search for FTN_1133 homologous was performed using the PSI-BLAST [22] and JACKHMMER [23] programs that ran against the non-redundant (nr) protein database of National Center for Biotechnology Information (NCBI). Secondary structure was predicted using the JPred program [24]. Alignment of sequences was performed using the Kalign algorithm [25] and processed by Jalview [26].

### Protein expression

The induction of recombinant FTN_1133 expression was achieved by adding 0.1 mM of isopropyl 1-thio-β-D-galactopyranoside (IPTG) in exponential culture (OD_600 nm_ = 0.5) of *E.coli* AD494 (*DE3*) harboring the pET15b-*FTN_1133* plasmid. Incubation was performed at 20°C with shaking and after 16 hours, cells were harvest by centrifugation and suspended in lysis buffer (10% glycerol, 500 mM NaCl, 20 mM sodium phosphate pH 7.4, 1 mM PMSF and 20 mM imidazole). Cells were disrupted by sonication (twelve alternating cycles of 15 seconds of sonication and 1 minute on ice bath) and debris was separated from supernatant by centrifugation at 15.000 rpm at 4°C for 40 minutes. The supernatant was filtered using a 0.45 µm pore membrane and applied using a peristaltic pump into HiTrap TALON crude column (GE Healthcare) containing agarose-Co^++^ resin. The column was washed sequentially with 3 column volumes of washing buffer (500 mM NaCl, 20 mM sodium phosphate pH 7.4) containing 50 mM and 100 mM of imidazole and eluted with 3 column volumes of elution buffer (500 mM NaCl, 20 mM sodium phosphate pH 7.4 and 500 mM of imidazole). Buffer exchange and concentration of purified proteins was done using Amicon Centrifugal 10 MW devices (Millipore). His-FTN_1133 purity was checked by SDS-PAGE analysis and the concentration was spectrophotometrically determined by its absorbance at 280 nm (ε**_280 nm_ = **11460 M^−1^ cm^−1^, according ProtParam tool [27]).

### Secondary structure determination

Circular Dichroism (CD) spectra were collected on a JASCO J-710 CD spectrometer using 3 or 6 µM of FTN_1133 in the presence of 100 mM NaCl, 20 mM sodium phosphate pH 7.4 and 10% glycerol in a 0.01 cm cell. Each CD spectrum resulted from the accumulation of 8 scans collected at scan rate of 20 nm/min with 1 nm band width and a time constant of 1 s. Data were collected from 260 to 195 nm and deconvoluted with CDpro-package software that uses SELCON3, CONTINLL, CDSSTR and CD CLUSTER algorithms [28]. Secondary structure prediction was performed with Jpred and a cartoon representation of secondary structure was draw with Polyview tool [29].

### Thiol dependent peroxidase activity assays

Reduction of cumene hydroperoxide (CuOOH), t-butyl hydroperoxide (tBOOH) or H_2_O_2_was monitored by FOX assay [30] using DTT, β-mercaptoethanol or glutathione as reducing agents. Briefly, FTN_1133 (10 µM), DTPA (Diethylene Triamine Pentaacetic Acid) (0.1 mM), sodium azide (0.1 mM), DTT (0.5 mM), β-mercaptoethanol (5 mM) or reduced glutathione (GSH) (5 mM) and CuOOH, tBOOH or H_2_O_2_ (200 µM) were mixed in 50 mM Hepes-HCl buffer, pH 7.4 (final volume of 0.1 mL) at 37°C during 0, 10, 20 and 40 minutes. Reactions were started by addition of hydroperoxides and stopped by addition of 20 µL of 5 M of HCl. Afterwards, reactions mixtures were diluted into 0.9 mL of FOX solution [250 µM of Fe^2+^ dissolved in 90% methanol, 25 mM of H_2_SO_4_, 4 mM BHT (Butylated Hydroxy Toluene) and 100 µM xylenol orange] and incubated during 30 minutes at 37°C. The absorbance values were recorded at 560 nm and concentrations were calculated according to calibration curve previously performed for all tested peroxides.

In the oxidized DTT assay, the peroxidase activity was assessed in the presence and absence of the Thioredoxin (TrxA) from *E.coli* (Sigma-Aldrich #T0910). Formation of oxidized DTT was followed by its intrinsic absorbance at 310 nm (ε_310 nm_ = 110 M^−1^.cm^−1^) [31], [32]. The reaction mixtures had a final volume of 1 mL and contained FTN_1133 (5 µM or 10 µM), TrxA from *E.coli* (1 µM), DTPA (1 mM) and CuOOH or tBOOH (2 mM) in 100 mM of sodium phosphate buffer, pH 7.4. Reactions were initiated by addition of DTT (10 mM).

Peroxidase activities were also analyzed by the lipoamide/Lipoamide dehydrogenase and GR/Grx/GSH system coupled assays. In these cases, rates were determined by decay of absorbance at 340 nm, as a consequence of NAD(P)H oxidation [21], [33] and [34].

For the lipoamide/Lipoamide dehydrogenase coupled assay, reaction mixtures of 1 mL (final volume) containing FTN_1133 (10 µM) or OsmC (10 µM) in the presence of reduced lipoamide (50 µM), DTPA (100 µM), Lpd (Dihydrolipoamide dehydrogenase from *X. fastidiosa*) (0.5 µM) and NADH (200 µM) in 50 mM of sodium phosphate buffer, pH 7.4, were initiated by addition of CuOOH, tBOOH or H_2_O_2_ (200 µM) [21].

For the GR/Grx/GSH coupled assay, reaction mixtures of 1 mL (final volume) contained FTN_1133 (5 µM), yeast Glutathione Reductase (GR) (6 µg/ml), Glutaredoxin C (GrxC) (10 µM), GSH (1 mM), bovine serum albumin (BSA) (0.1 mg/ml), DTPA (2 mM) and NADPH (0.2 mM) in 100 mM Tris–HCl buffer, pH 7.4. Reactions were initiated by addition of CuOOH or tBOOH (0.2 mM). GR and GrxC activities were previously assayed by their capacity to reduce the mixed disulfide between HED (2-Hydroxy Ethyl Disulfide) (0.7 mM) and GSH, when incubated at 30°C for 3 min. Reactions were started by the addition of GrxC and followed by the decrease in the absorbance at 340 nm due to the oxidation of NADPH [33].

For FOX and peroxidase-coupled lipoamide assays, we included a recombinant OsmC protein as positive control, since this Cys-based peroxidase displays activity against organic hydroperoxide using DTT and lipoamide as reducing systems [21].

### Functional complementation assay

Two mutant strains of *E. coli* were chosen for complementation analysis: (i) an *ahpC E. coli* BW25113 mutant, which was deficient in response to organic hydroperoxide and (ii) an *oxyR E.coli* BW25113 mutant, which was deficient in response to H_2_O_2_ and to a lower extent to organic hydroperoxide challenge, as assessed by the disk inhibition assay (unpublished data).

To test if FTN_1133 was capable to complement the organic peroxide sensitive phenotype, mutant strains that harbored pPROEX expression vectors containing *FTN_1133* or *osmC* (positive control) genes were grown until OD_600 nm_ = 0.5, induced by addition of 0.5 mM of IPTG for two hours and plated on LB supplemented with ampicillin (100 µM) and IPTG (100 µM). After that, 6 mm paper filter disks were saturated with 10 µL of solution of tBOOH (75 mM), CuOOH (100 mM) or H*_2_*O_2_ (500 mM) and placed on the surface of inoculated LB plates. After 16 hours, the size of inhibition zone was measured with a ruler. The statistical analyses were performed between wild type and mutant strains carrying the pPROEX-*FTN_1133* plasmid and analyzed by the unpaired Student's t-test. In each graph, error bars represent the standard deviation (n = 9).

### pK_a_ determination of single FTN_1133 cysteine residue

The pK_a_ of the cysteine residue from FTN_1133 was determined by a monobromobimane (mBrB) alkylation method that generates a fluorescent product, detected at λ_exc_ 396 nm and λ_em_ 482 nm [35]. FTN_1133 was pre-reduced with DTT (100 mM) for 16 hours at 4°C in the presence of NaCl (500 mM) and sodium phosphate buffer, pH 7.4 (20 mM). Excess of DTT was eliminated by size-exclusion chromatography (PD-10 column, GE HealthCare) with NaCl (500 mM). Reduced FTN_1133 (4 µM) was then incubated with monobromobimane (8 µM) in the presence of 15 mM of acetic acid,15 mM of MES [2-(N-Morpholino Ethanesulfonic Acid] and 30 mM of Tris-HCl buffer adjusted to the pH values from 3.5 until 9.0. The assays were performed in flat-bottom white polystyrene 96-well plates (Costar) as triplicates, using Eclipse Varian Spectrofluorimeter, operating at medium voltage with both emission and excitation slit of 5 nm. As control of method, the same procedure was performed with recombinant Ohr protein from *Xylella fastidiosa*, and OsmC protein from *E. coli*. An additional blank reaction was performed in absence of thiols, to determine whether buffer components could interfere with the reaction or not.

The angular coefficients were calculated using time points that included at least the initial 10 min of reaction that were fitted in a straight line. The plots displayed were fitted by non-linear regression to Henderson-Hasselbalch equation considering 95% of confidence using Prism 4 for Windows, GraphPad Software, San Diego, CA.

### Inter-molecular disulfide bond formation in FTN_1133 evaluated by non-reducing SDS-PAGE

Induction of inter-molecular disulfide bond in FTN_1133 by organic or by H_2_O_2_ was analyzed by non-reducing SDS-PAGE. Aliquots of FTN_1133 (5 µM) were treated with 1, 5, 10, 20 50 or 100 µM CuOOH, tBOOH or H_2_O_2_ in a buffer containing NaCl (0.1 M) and DTPA (1 mM) in 20 mM of sodium phosphate buffer, pH 7.4, for ten minutes at 37°C. Immediately after that, samples were treated with 100 mM with NEM (N-Ethyl Maleimide) for one hour at room temperature.

In another experiment, aliquots of FTN_1133 (5 µM) were treated with CuOOH, tBOOH or H_2_O_2_ (100 µM) in a sodium phosphate (20 mM) buffer, pH 7.4 containing NaCl (0.5 M) and DTPA (1 mM) for 0, 0.5, 1, 2, 3, 4, 18 and 22 hours at 30°C and after each time point, the remaining cysteine sulfhydryl groups were alkylated with NEM (100 mM) for one hour at room temperature. As control of experiment, aliquots of FTN_1133 (5 µM) were submitted to the same experimental conditions, with no addition of hydroperoxides.

After all treatments, the respective samples were separated in a 14% non-reducing SDS-PAGE gel and stained by Comassie Blue.

## Results

### FTN_1133 did not display features of OsmC/Ohr proteins or other thiol-dependent peroxidases

**Figure 1:**
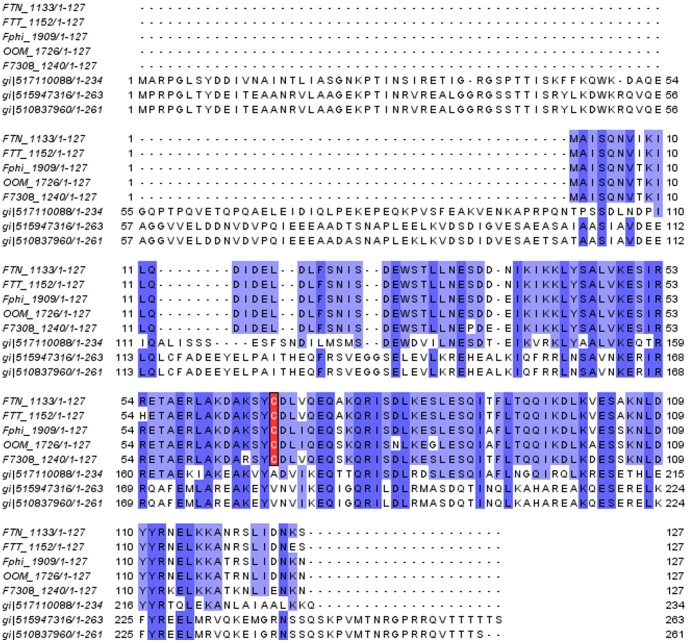
Sequence alignment of FTN_1133. FTN_1133 sequence was run against non-redundant database of NCBI using PSI-Blast algorithm operating in a default mode. The alignment of sequences from PSI-Blast output was generated by using the Kalign algorithm and processed by Jalview. The location of the cysteine residue on *Francisella* sequences is indicated by red boxes. Dark and light blue shadings backgrounds illustrate regions with greater than 80% of similarity presented among all sequences. Protein sequence ID FTN_1133 from *F. tularensis novicida* U112 has 100% amino acid identity with FTE_0530 (*F. tularensis novicida* FTE), FTG_0533 (*F. tularensis novicida* FTG), FN3523_1168 (*F. tularensis novicida* 3523) and FNFX1_1179 (*F. tularensis novicida* Fx1). Protein sequence IDFTT_1152 from *F. tularensis tularensis SCHU4* has 100% identity with FTL_0803 (*F. tularensis holarctica* LVS), FTF_1152, (*F. tularensis tularensis* FSC198), FTH_0797 (*F. tularensis holarctica* OSU18), FTW_1191 (*F. tularensis tularensis* WY96_3418), FTA_0849 (*F. tularensis holarctica* FTNF002_00), FTM_0836 (*F. tularensis mediasiatica* FSC147), FTU_1185 (*F. tularensis tularensis* TIGB03), FTV_1101 (*F. tularensis tularensis* TI0902), FTS_0796 (*F. tularensis holarctica* FSC200) and FTHG_00746 (*F. tularensis holarctica* 257). Protein sequences IDOOM_1726, Fphi_1909 and F7308_1240 are from *F. noatunensis orientalis* str. Toba 04, *F. philomiragia philomiragia* ATCC 25017 and *Francisella sp.* TX077308, respectively. Protein sequence ID, gi|517110088 is from *Fangia hongkongensis* and protein sequences ID, gi|515947316 and gi|510837960, are from *Piscirickettsia salmonis*.

FTN_1133 is a protein with 127 amino acid residues and, in contrast to the Ohr/OsmC proteins (which depends on two cysteine residues for its peroxidase activity), has only one cysteine residue (Fig. 1). FTN_1133 primary sequence also lacks PXXX(T/S)XXC, the canonical motif that are present in all proteins belonging to the peroxiredoxin family [36].

Additional search in the pFAM database did not retrieve any hits, using FTN_1133 sequence as query. In another approach, only hits of genes/proteins from close relatives to *Francisella tularensis* were retrieved (Fig. 1).The three proteins present in *Fangia hongkongensis* and *Piscirickettsia salmonis* have an additional domain named KfrA at their N-terminal portion, which are postulated to be involved with plasmid replication but without a precise role assigned [37]. FTN_1133 amino acid sequence shares similarity with the C-terminal domain, having no homology with the KfrA domain. Remarkably, a Cys residue is conserved among *Francisella* FTN_1133 related sequences but not in proteins containing the KfrA domain.

It is worthwhile to mention that a PSI-blast search using FTN_1133 sequence as query, retrieved one sequence of OhrA from *Bacillus megaterium*. However, this alignment presented a low e-value (0.64) and poor coverage (37%). The lack of significance between FTN_1133 and Ohr family was corroborated by performing a PSI-blast analysis using OhrA sequence from *Bacillus megaterium* as query. This inverted analysis did not retrieve FTN_1133 sequence, even after 10 iterations (data not shown). Additionally, FTN_1133 sequence was not present among 7485 sequences belonging to the Ohr/OsmC family in the pFAM database.

Therefore, it is not appropriate to consider FTN_1133 a member of the OsmC/Ohr family. We characterized the recombinant FTN_1133 structural and biochemically in attempt to understand its possible function as a thiol peroxidase.

### FTN_1133 secondary structure analysis

**Figure 2:**
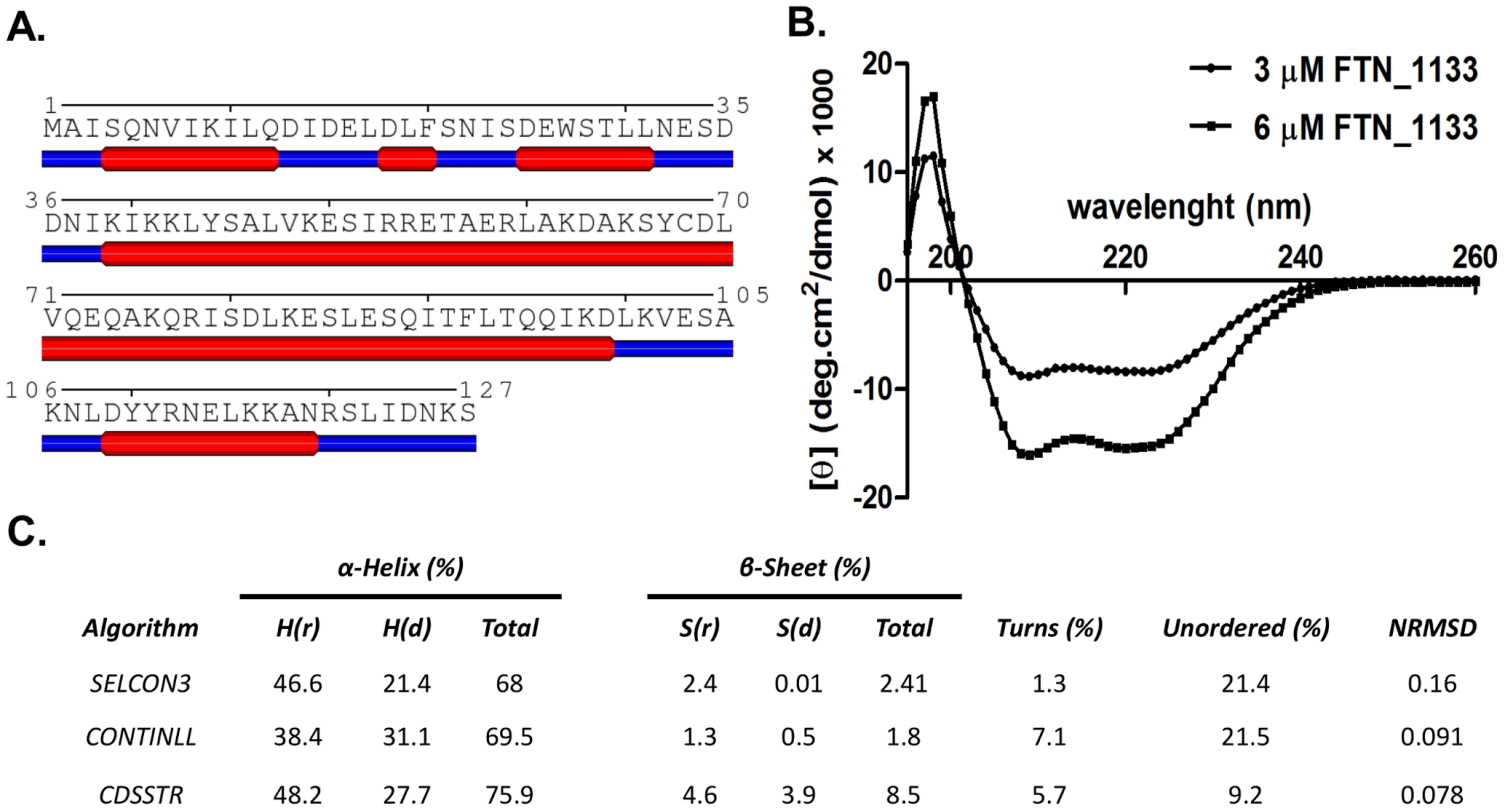
FTN_1133 secondary structure. **A.** Cartoon of predicted FTN_1133 secondary structure. Blue traces represent predicted loop regions and red traces represent predicted α-helix region of FTN_1133 sequence. **B.** Circular dichroism spectra of FTN_1133 were collected with 3 and 6 µM in the presence of NaCl (100 mM), sodium phosphate pH 7.4 (20 mM) and 10% glycerol using a 0.01 cm cell in a JASCO J-710 spectropolarimeter. **C.** Estimates of secondary structure elements of FTN_1133 from data obtained from item B., using SELCON3, CONTINLL, CDSSTR algorithms. NRSMD, denotes the Normalized Root-Mean-Square Deviation.

Analysis by Jpred prediction tool suggested that FTN_1133 is an all-α- helix protein (Fig. 2A). To validate the ‘*in silico’* prediction, we performed circular dichroism analysis of FTN_1133 recombinant protein. CD spectra of FTN_1133 display minima at 208 nm and 222 nm, characteristic of proteins that have only α-helix and not β-sheet (Fig. 2 B). Indeed, deconvolution of FTN_1133 CD spectra carried out with CDPro software, revealed a negligible β-sheet content independent of the algorithms used (Fig. 2 C). So, CD analysis confirmed the ‘*in silico*’ prediction, and this result contrasts to what was observed for peroxiredoxin and Ohr/OsmC enzymes both of them containing a considerable amount of β-sheet.

### FTN_1133 did not present thiol dependent peroxidase activity *in vitro*

**Figure 3:**
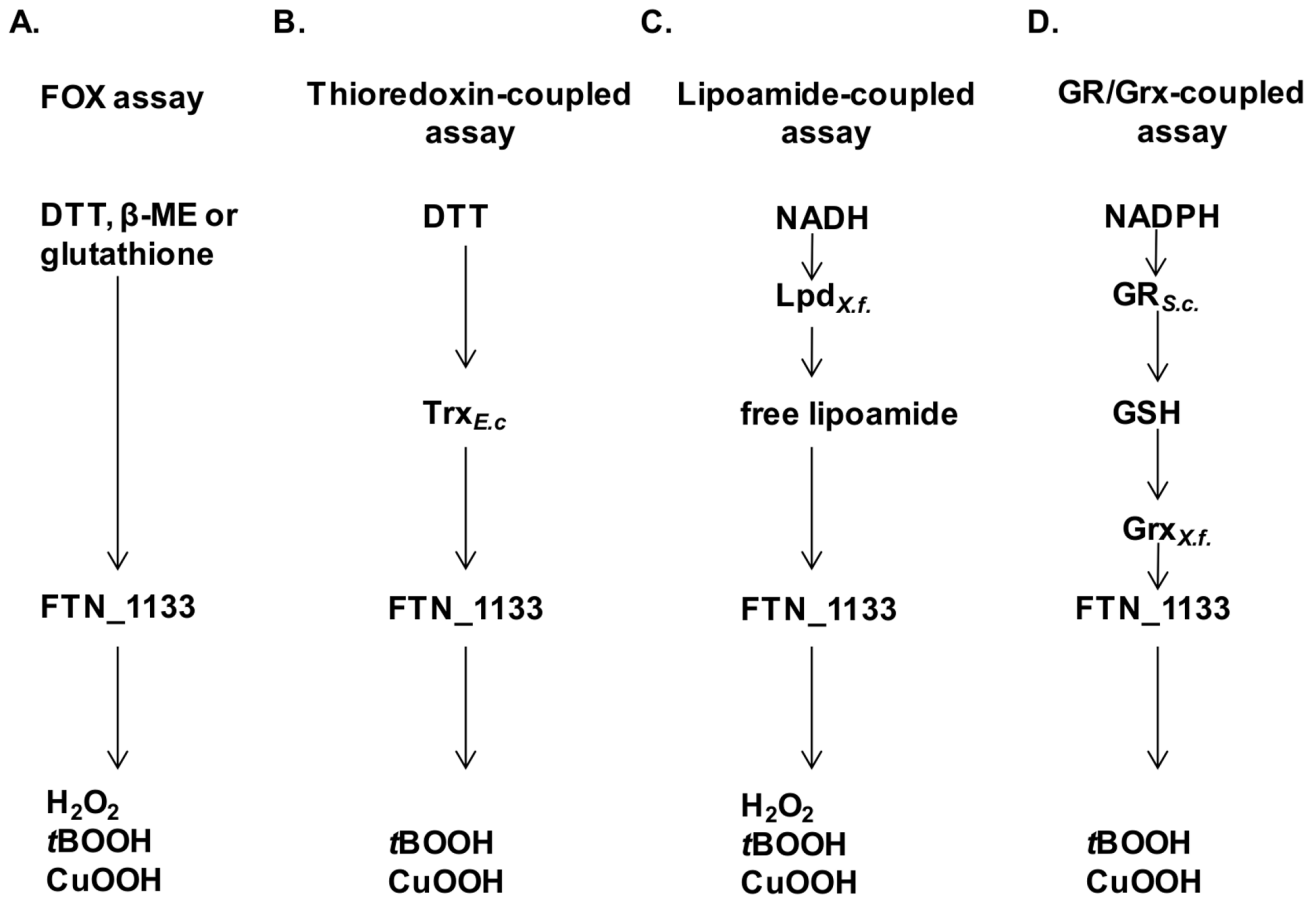
Squematic electron flow of systems that were used to test a possible thiol-dependent peroxidase activity of recombinant FTN_1133. **A.** Consumption of hydroperoxides determined by FOX assay, using DTT, β-mercaptoethanol or glutathione as electron donors. **B.** Oxidized DTT assay was used to monitor the ability of thioredoxin to support a putative FTN_1133 activity. In this case, thioredoxin would provide electrons to reduce FTN_1133 instead of DTT, which would be engaged to recycle the thioredoxin protein; **C.** For lipoamide-coupled assay, electrons originated from NADH are transferred to a Dihydrolipoamide dehydrogenase (Lpd), that through lipoamide would reduce FTN_1133; **D.** In the GR/Grx-coupled assay, electrons originated from NADPH would flow to FTN_1133, through GR/Grx system. β-ME, β-mercaptoethanol; Trx_E.c._, Thioredoxin from *E. coli*; LpD_X.f._, Dihydrolipoamide Dehydrogenase from *X. fastidiosa*; GR_S.c._, Glutathione Reductase from baker's yeast *S. cerevisae*; GrxC_X.f._, Glutaredoxin C, from *X. fastidiosa*.

So far, our studies revealed that FTN_1133 protein displays characteristics that differ from Ohr/OsmC family and other known thiol dependent peroxidase enzymes. However, previously it was shown that *Francisella tularensis subsp. novicida* cells deficient on FTN_1133 present lower capacity to decompose organic peroxides [16]. So, we tested if recombinant FTN_1133 is a thiol peroxidase, employing various assays based on electron flows summarized in Figure 3.

**Figure 4:**
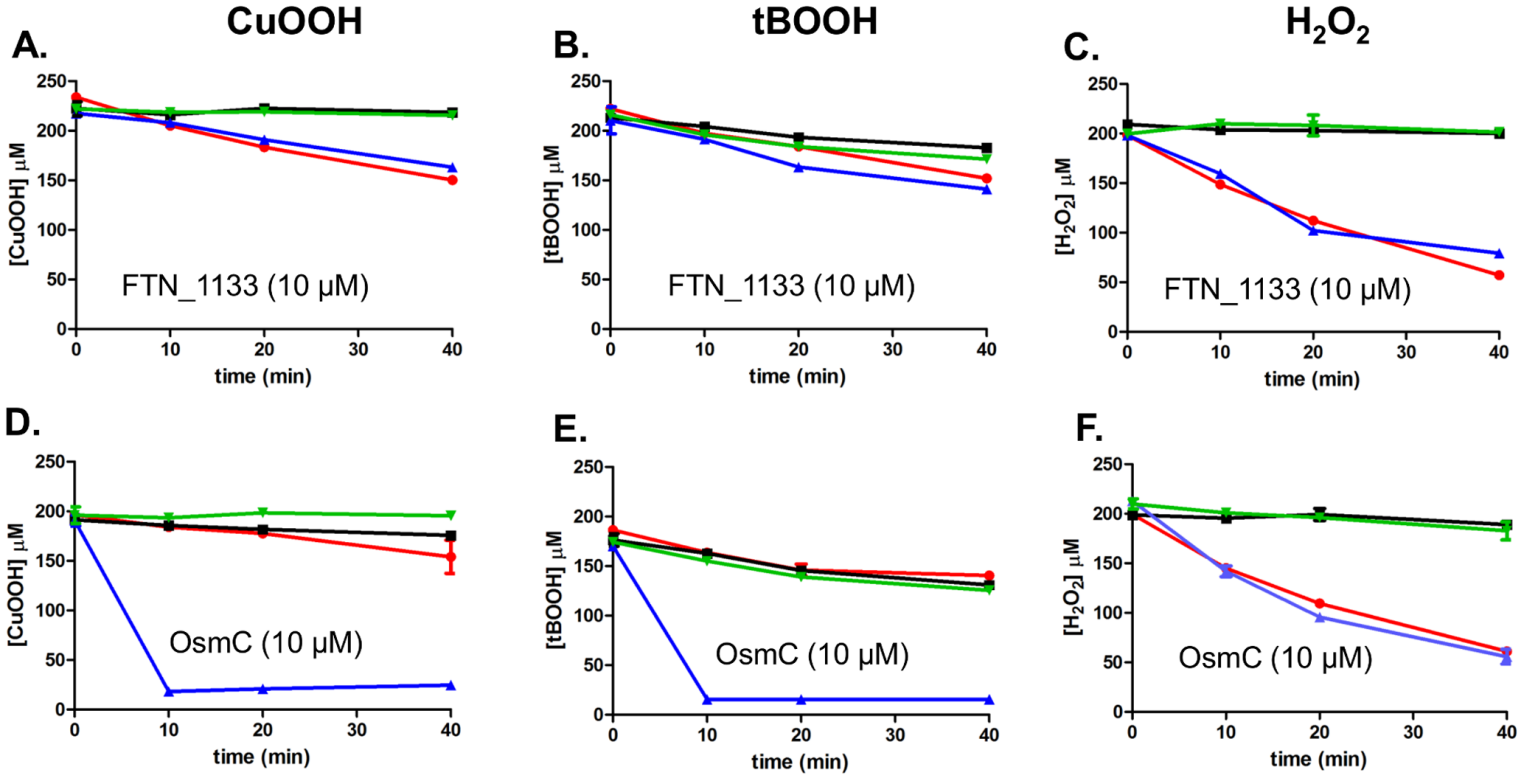
Assay for FTN_1133 DTT-dependent peroxidase activity. Decomposition of different peroxides was monitored during 40**A.**, **B.** and **C.**, CuOOH, tBOOH and H_2_O_2_ decomposition, respectively, in the presence of FTN_1133 (10 µM), HEPES-HCl pH 7.4 (50 mM), DTT (0.5 mM), sodium azide (0.1 mM) and DTPA (0.1 mM). **D.**, **E.** and **F.**, CuOOH, tBOOH and H_2_O_2_ decomposition, respectively, in the presence of OsmC (10 µM), HEPES-HCl pH 7.4 (50 mM), DTT (0.5 mM), sodium azide (0.1 mM) and DTPA (0.1 mM). All reactions were started by addition of 200 µM of peroxide. Blue line, (blue triangle) enzyme+DTT+peroxide (catalyzed reaction); Green line, (green triangle) enzyme+peroxide without DTT; Black line, (black square) only peroxide and red line, (red circle) peroxide+DTT (uncatalyzed reaction). The figure is representative of at least two independent set of experiments, each one done in technical triplicates.

We first measured the amount of hydroperoxide (CuOOH, tBOOH or H_2_O_2_) that remained in the reaction mixtures, after incubation of substrate with FTN_1133 in the presence of DTT. FTN_1133, even at high concentrations, was not able to degrade CuOOH or tBOOH faster than the uncatalyzed reaction (absence of protein), even after 40 minutes of reaction (Fig. 4 A and B, respectively). In comparison, the same amount of OsmC was able to decompose, almost completely CuOOH and tBOOH in the first 10 minutes of reaction (Fig. 4 D and E, respectively). Both FTN_1133 and OsmC, did not presented activity against H_2_O_2_ (Fig. 4 C and F, respectively). This was expected for OsmC, since this enzyme had been reported to present reduced capacity to detoxify H_2_O_2_ when compared to organic hydroperoxides [20]. Furthermore, FTN_1133 was also not able to reduce hydroperoxides when monothiols, β-mercaptoethanol (5 mM) or glutathione (5 mM) were used as reducing agents instead of DTT, as evaluated by FOX assay (data not shown).

Since during oxidation/reduction cycle of enzyme, the reduction by DTT or by monothiols could be the rate-limiting step, we tried to use alternative reductive systems based on thioredoxin, lipoamide and GR/Grx/GSH (Fig. 3). However, FTN_1133 did not display any measurable peroxidase activity, even using alternative reducing systems, distinct hydroperoxides and distinct assays (Fig. S1, S2 and S3). These results indicate that FTN_1133 does not display a peroxidase activity, but we cannot exclude the possibility that it can indirectly remove these oxidants. It is also possible that some yet unidentified component in the *Francisella tularensis* cell might be required to confer ability to FTN_1133 to decompose organic hydroperoxides.

### Functional complementation assay

The *in vitro* assays presented so far indicated that FTN_1133 did not display thiol dependent activity no matter the reducing system employed. Then, we decided to test if heterologous expression of FTN_1133 would be able to restore wild-type hydroperoxide susceptibility presented by *E. coli ahpC* and *oxyR* mutant strains. For this purpose, *FTN_1133* and *osmC* (positive control) genes were cloned into pPROEX expression vector. The expression of the recombinant proteins FTN_1133 and OsmC in *E. coli ahpC* and *oxyR* mutant strains were checked by *western blot* (Fig. S4). Then, the capacity of the expression vectors to complement the hydroperoxide sensitive phenotype was evaluated by disk inhibition assay.

**Figure 5:**
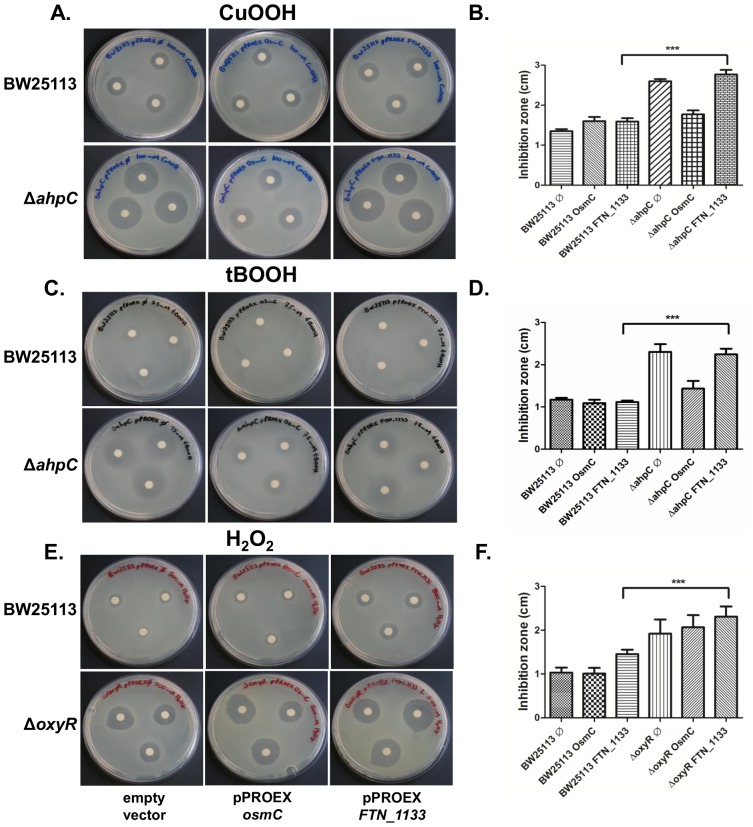
Complementation assay. Recombinant FTN_1133 was expressed in *E. coli* mutant strains in attempts to complement their hypersensitivity phenotype. **A.** CuOOH disk inhibition assay for *E. col*i BW25113 (wild type) and *ahpC* mutant strains that harbor empty pPROEX, pPROEX_*FTN_1133* or pPROEX_*osmC* constructions; **B.** Statistical analysis from data described in A, *** *p*<0.001 as determined by the Student's t-test. **C.** tBOOH disk inhibition assay for *E. col*i BW25113 (wild type) and *ahpC* mutant strains that harbor empty pPROEX, pPROEX_FTN_1133 or pPROEX_OsmC constructions; **D.** Statistical analysis from data described in C, *** p<0.001. **E.** H_2_O_2_ disk inhibition assay for *E. col*i BW25113 (wild type) and *oxyR* mutant strains that harbor empty pPROEX, pPROEX_FTN_1133 or pPROEX_OsmC constructions; **F.** Statistical analysis from data described in C, *** *p*<0.001. In each graph, error bars represent the standard deviation (n = 9).

Wild type strain that harbors an empty copy of expression vector, presented halo sizes of 1.35±0.05 and 1.17±0.04 cm, when treated with paper filters immersed on 100 mM of CuOOH (Fig. 5 A and 5 B) or 75 mM of tBOOH (Fig. 5 C and 5 D), respectively. When treated with CuOOH or tBOOH, the *ahpC* mutant strain that harbors an empty copy of expression vector, presented halo sizes of 2.60±0.05 and 2.3±0,18 cm, respectively, while the same strain that over-expressed FTN_1133 presented inhibition zones of 2.76±0.11 and 2.24±0.13 cm. These results indicate that overexpression of FTN_1133 did not restore the wild type phenotype. In contrast, when OsmC was over-expressed in *ahpC* mutant strain, the sizes of the halos were 1.77±0.98 cm for CuOOH treatment (Fig. 5 A and 5 B) and 1.43±0.18 for tBOOH treatment (Fig. 5 C and 5 D). Therefore, OsmC over-expression rescued the wild type phenotype. In addition, the expression of FTN_1133 and OsmC did not increase the resistance to H_2_O_2_ of the *oxyR* mutant. Expression of FTN_1133 (inhibition zone of 2.30±0.23 cm) or OsmC (2.06±0.27 cm) (Fig. 5 E and F) resulted in similar growth of bacteria carrying empty plasmid (inhibition zone of 1.92±0.32 cm). Again, our results indicated that recombinant FTN_1133 does not present peroxidase activity.

### Determination of FTN_1133 thiolate pK_a_

All the Cys-based thiol peroxidases so far described carry reactive cysteine, whose thiolate groups display an acidic pK_a_ that is related with the stabilization of the transition state [38], [39]. Therefore, we decided to determine the pK_a_ value of the single Cys residue of FTN_1133 by the monobromobimane alkylation method [35].

**Figure 6:**
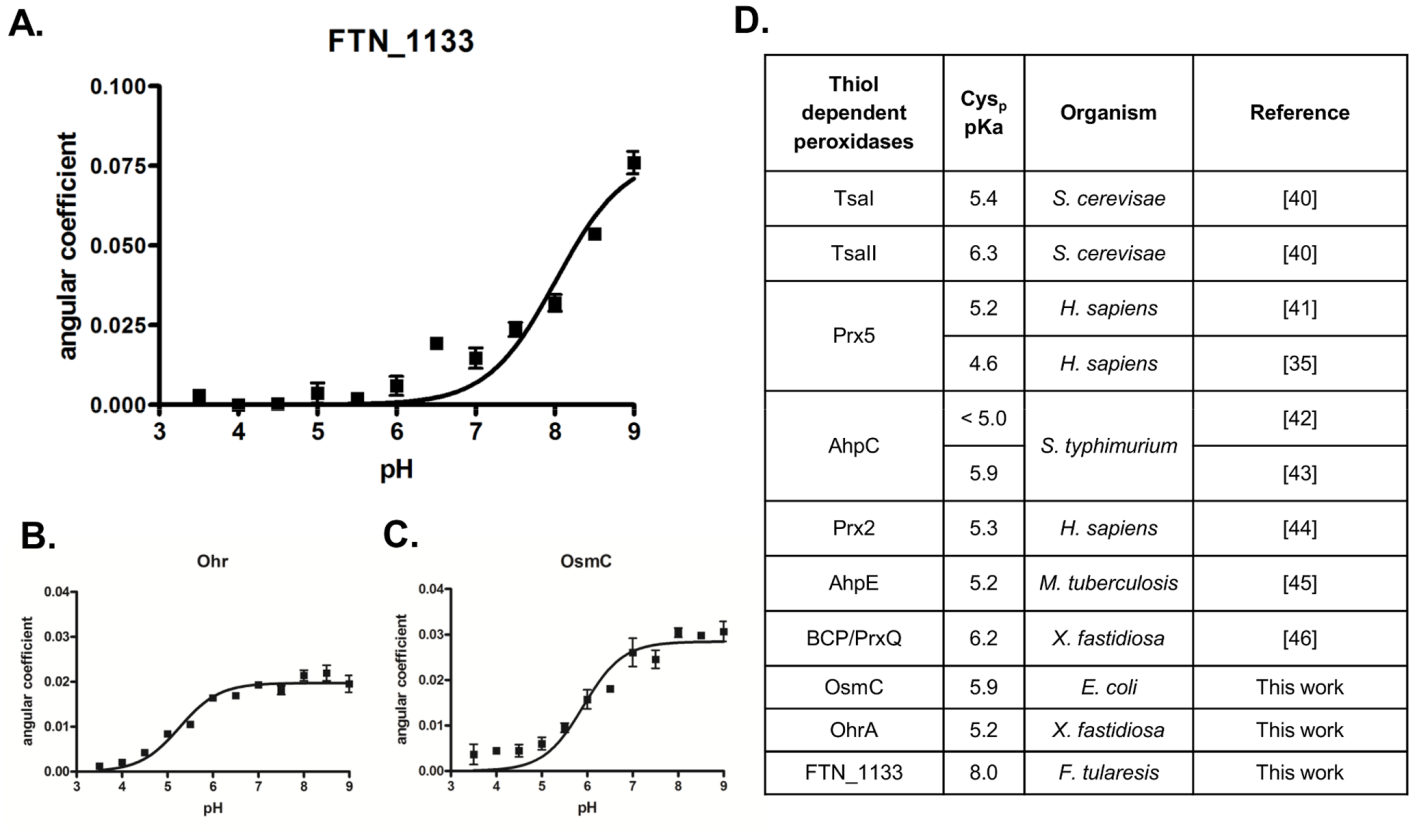
Determination of pK_a_ values for thiolate groups. pKa values were determined by the ability of monobromobimane to alkylate preferentially thiolate (RS^−^) over thiol (RSH) groups [35]. Monobromobimane alkylation was performed with pre-reduced protein (4 µM) and monobromobimane (8 µM) in the presence of acetic acid (15 mM), MES (15 mM) and Tris-HCl (30 mM) buffer adjusted to pH values from 3.5 to 9.0. **A.** The determined pKa value of FTN_1133 thiolate was 8.0 (±0.12) and ranged from 7.7 to 8.2, if it is considered 95% as the confidence interval of the best-fit curve. **B.** and **C.** The determined peroxidatic cysteine (Cys_p_) pKa values of Ohr and OsmC using the monobromobimane method were 5.27 (±0.07) and 5.9 (±0.1), respectively. The pK_a_ value ranged from 5.11 to 5.41 for Ohr or 5.68 to 6.13 for OsmC, again in the 95% confidence interval. The figure is representative of two independent set of experiments. **D.** Cys_p_ pKa values of selected thiol-dependent peroxidases, whose peroxidase activities have been experimentally demonstrated.

Angular coefficients were proportional to the rate of FTN_1133 cysteine alkylation by monobromobimane at different pHs (Fig. 6 A) and the curve that best fitted the experimental data by non-linear regression (Handerson-Hasselbach equation) indicated pKa value of 8.0±0.1 for the thiolate group of FTN_1133. If we consider the 95% confidence interval, the FTN_1133 apparent pK_a_ value ranged from 7.7 to 8.2, which can be considered high when compared to values observed for reactive Cys residues from proteins whose thiol dependent peroxidase activities are well established (Fig. 6 D), [35], [40], [41], [42], [43], [44], [45] and [46]. In fact, when the same approach was carried out for the recombinant Ohr and OsmC proteins, the pK_a_ values were 5.3±0.07 and 5.9±0.1, respectively (Fig. 6 B and C).

### FTN_1133 inter-molecular disulfide bond formation in response to hydroperoxide treatment

Since we observed that a fraction of purified FTN_1133 migrated as a dimer in non-reducing SDS PAGE (data not shown), we decided to study the thiol redox state of its cysteine residue in response to peroxides based on previously published methods [47] and [48].

**Figure 7:**
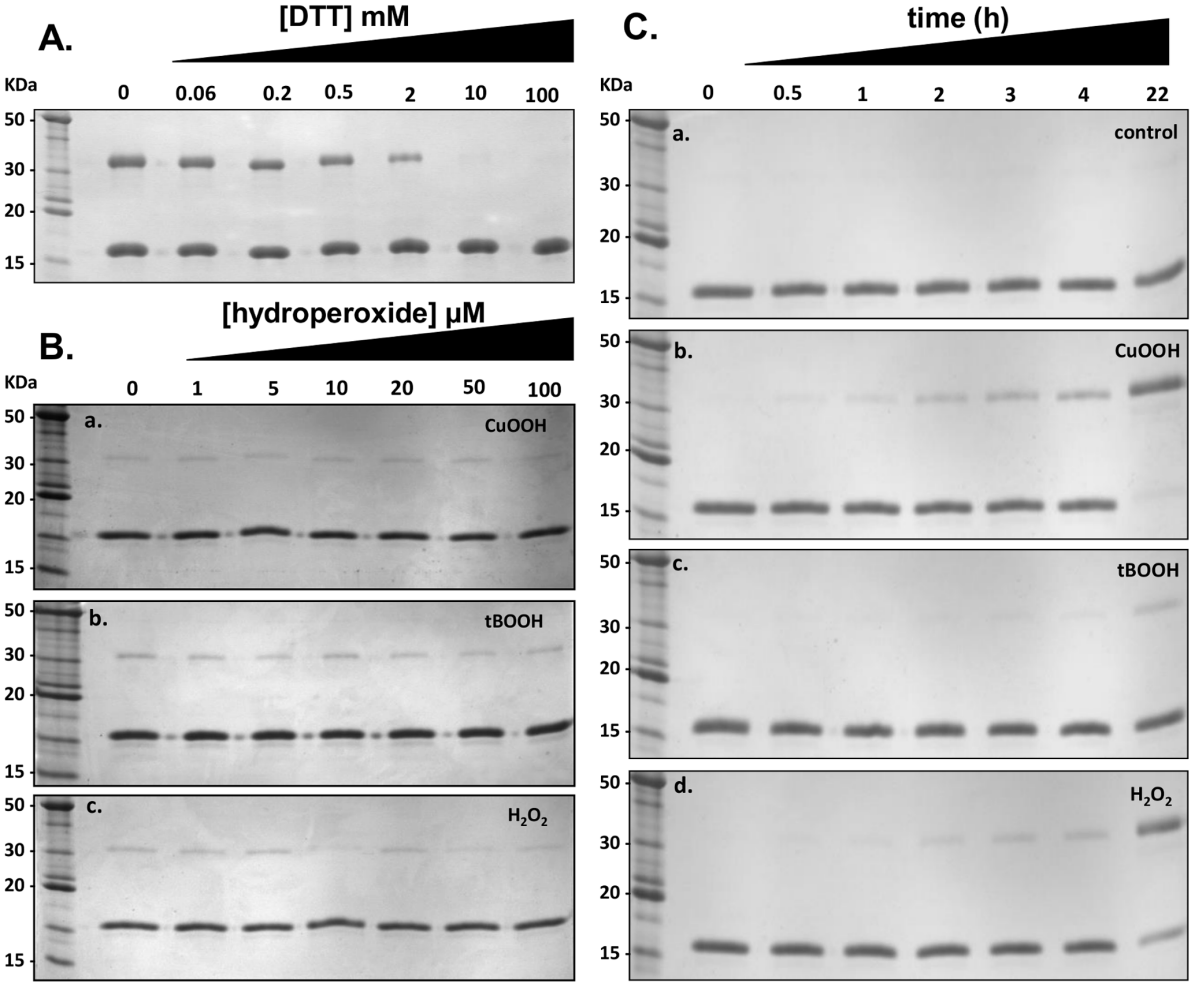
Inter-molecular disulfide bond formation in FTN_1133 upon hydroperoxide treatment. A. Non-reducing SDS-PAGE of freshly purified recombinant FTN_1133 (5 µM) that was incubated in the absence or presence of 0.06, 0.2, 0.5, 2, 10 and 100 mM of DTT during 10 minutes at 37°C. **B.** Representative non-reducing SDS-PAGE showing that the treatment of FTN_1133 with increasing amount of hydroperoxides. FTN_1133 inter-molecular disulfide bond formation was assessed by the appearance of a band corresponding to the dimer (∼34 kDa). Treatments, using pre-reduced FTN_1133 (5 µM), were performed for 10 minutes at 37°C with 0, 1, 5, 10, 20, 50 and 100 µM of CuOOH (a), tBOOH (b) or H_2_O_2_ (c). **C.** Time course of FTN_1133 oxidation towards hydroperoxide treatment. The assay was carried out with pre-reduced FTN_1133 (5 µM) treated with 100 µM CuOOH (b), tBOOH (c) or H_2_O_2_ (d), during 0, 0.5, 1, 2, 3, 4 and 22 hours at 30°C in a buffer containing 0.5 M of NaCl, 20 mM of sodium phosphate pH 7.4 and 1 mM of DTPA. (a) Represent the control reaction (no addition of hydroperoxide). Immediately after hydroperoxides treatments, all samples were alkylated with NEM (100 mM) for 30 minutes at room temperature to avoid oxidation artifacts due to protein denaturation by SDS. The figure is representative of three independent set of experiments.

First, we verified if the FTN_1133 dimer, which corresponds to an inter-molecular disulfide bond, was not an artifact generated during electrophoresis by denaturation of the protein by SDS [49]. For this purpose, purified FTN_1133 was previously blocked with NEM before loading the sample into a non-reducing SDS-PAGE. A band corresponding to the dimer (intermolecular disulfide bond) was still present (data not shown), indicating that the inter-molecular disulfide bridge formation was not an artifact. Then, FTN_1133 samples were reduced with increasing amounts of DTT, and a band corresponding to a FTN_1133 dimer decreased in intensity proportionally to amount of DTT applied (Fig. 7 A). Therefore, it was possible to investigate the oxidation of FTN_1133 in a way independent of the reducing system, i.e., by following inter-molecular disulfide formation upon hydroperoxide treatment. In this regard, reduced FTN_1133 (5 µM) was treated with 1, 5, 10, 20, 50 or 100 µM CuOOH, tBOOH or H_2_O_2_ for ten minutes at 37°C and no inter-molecular disulfide bond formation was observed (Fig. 7 B).

In a time-dependent investigation, FTN_1133 was exposed to 100 µM of CuOOH, tBOOH or H_2_O_2_ during different intervals and oxidation was only detected at very prolonged intervals (22 hours), when compared with the control reaction, where no hydroperoxide was added (Fig. 7 C).These results showed that inter-molecular disulfide formation of FTN_1133 was very slow upon induction by hydroperoxides. Visual inspection of gels, analyzing the band corresponding to the dimeric form of FTN_1133 (∼34 KDa), showed that the most efficient oxidant tested was CuOOH, followed by H_2_O_2_ and tBOOH (Fig. 7 C, a, b, c and d). This observation became evident by analysis of lane that corresponds to 22 hours of treatment. For CuOOH treatment, almost all monomeric form disappeared, while for tBOOH treatment, most of protein displayed a monomeric form and for H_2_O_2_ treatment, intermediary monomeric band intensity was detected at 22 hour of treatment.

As conclusion, oxidation of FTN_1133 by hydroperoxides were very slow, since even after four hours and using a large excess (1∶50) of CuOOH, tBOOH or H_2_O_2_, most of protein remained as a monomeric form in non-reducing gels, corresponding to the reduced state of FTN_1133.

## Discussion

*Francisella tularensis subsp. novicida* mutant for FTN_1133 gene presented a reduced capacity to detoxify organic hydroperoxides *in vivo* [16]. As a consequence, it was suggested that FTN_1133 could be a member of the Ohr/OsmC family. However, the results presented in this work strongly suggest that FTN_1133 is not a Cys-based thiol dependent peroxidase. Indeed, taking into account the data on Figure 7 C, we estimated that the second order rate constant between FTN_1133 and CuOOH is 0.1 M^−1^ s^−1^, that is several orders of magnitude lower than similar rate constants (10^6^–10^8^ M^−1^ s^−1^) of other thiol peroxidases [48], [50] and [19]. However, we cannot exclude the possibility that a post translational modification could be required for FTN_1133 to exhibit this enzymatic activity, since an increasing number of post translational modifications in bacteria have been described [51].

Taken together, the observed structural and biochemical features presented here for FTN_1133, contrast with those presented for other known thiol dependent peroxidases, including proteins belonging to the Ohr/OsmC family. Since orthologous proteins to FTN_1133 are restricted to *Francisella* genus, we raised the hypothesis that FTN_1133 could indirectly participate in a particular peroxidase system restricted to bacteria belonging to the *Francisella* genus. In support to this notion, AhpD (a thioredoxin-like protein) provides electrons to AhpC, a Cys based peroxidase from *Mycobacterium tuberculosis* [34]. However, FTN_1133 does not present a NXCXXC motif characteristic of thiol-reducing proteins [52]. In fact, performing a brief inspection into genomic context around the *FTN_1133* gene, we observed the occurrence of two neighborhood genes *FTN_1132* and *FTN_1134*, whose functions were not assigned yet. FTN_1132 was annotated as a hypothetical protein and its occurrence is restricted to *Francisella* genus (like FTN_1133 does). FTN_1134 presents a conserved Sdh5 domain that is involved in flavinylation and activation of Succinate Dehydrogenase (SDH) complex in both eukaryotes and bacteria [53] and [54]. Since at least one product of these two genes binds to FAD (Flavin Adenine Dinucleotide), a redox active molecule, it is tempting to speculate that FTN_1134 could be an electron donor required for a peroxidase system that FTN_1133 might participate.

FTN_1133 was clearly shown to be implicated with *Francisella* virulence [16], however more studies are required to fully understand its involvement in the response of bacteria to oxidative stress.

## Supporting Information

Figure S1 **Assay for FTN_1133 thioredoxin-dependent peroxidase activity.** Peroxidase activity was followed by generation of oxidized DTT. **A.** and **B.** DTT oxidation by CuOOH or tBOOH, respectively. Reactions were carried out in the presence of FTN_1133 (5 µM), sodium phosphate pH 7.4 (100 mM), DTPA (1 mM) and organic hydroperoxide (2 mM), with (red line) or without (green line) addition of 1 µM of recombinant TrxA from *E. coli*, and started by addition of 10 mM of reduced DTT. As positive control of reaction, the same assay was made using 10 µM of OsmC without addition of TrxA (blue line). The blank reaction (black line) was performed without any enzyme addition. The figure is representative of at least two independent set of experiments.
(TIF)

Figure S2 **Assay for FTN_1133 lipoyl-dependent peroxidase activity.** Lipoamide-Lipoamide dehydrogenase coupled assay was followed decay of absorbance at 340 nm due to NADH oxidation. **A.**, **B.** and **C.**, represent NADH oxidation in the presence of CuOOH, tBOOH and H_2_O_2_ at 37°C, respectively. Reactions were performed with FTN_1133 (10 µM), sodium phosphate pH 7.4 (50 mM), of reduced lipoamide (0.05 mM), DTPA (1 mM), recombinant Lpd (0.005 mM) from *Xyllela fastidiosa* under gently agitation (red line). After one minute for temperature stabilization, 0.2 mM of NADH was added to reaction that was finally started by addition of 0.2 mM of respective hydroperoxide. As positive control of reaction (blue line), the same assay was carried out using 10 µM of OsmC. The blank reaction (black line) was performed without any enzyme addition. The figure is representative of at least two independent set of experiments.
(TIF)

Figure S3 **Assay for FTN_1133 Grx/GSH-dependent peroxidase activity.** GR/GSH coupled assay was followed by NADPH oxidation. **A.** and **B.**, NADPH oxidation in the presence of CuOOH and tBOOH at 37°C, respectively. The reaction containing Tris–HCl pH 7.4 (100 mM), yeast GR (6 µg/ml), GrxC (10 µM) from *X. fastidiosa*, GSH (1 mM), BSA (0.1 mg/ml), DTPA (2 mM) and NADPH (0.2 mM) was initiated by addition of 0.2 mM of CuOOH or tBOOH. **C.** For GR and GrxC activity control reaction, FTN_1133 and hydroperoxide, were substituted by 0.7 mM of HED that was incubated at 30°C for 3 min for the formation of the mixed disulfide between GSH and HED. The reaction was started by the addition of GrxC*_x.f._* and followed by the decrease in the absorbance at 340 nm due to the oxidation of NADPH [33]. The figure is representative of at least two independent set of experiments.
(TIF)

Figure S4 **Expression analysis of recombinant FTN_1133 and OsmC proteins in wild type (BW25113), Δ** ***ahpC*** **and Δ** ***oxyR*** **backgrounds.** **A.** and **C.** Comassie stained gels of total extracts from IPTG induced cultures of wild type BW25113 (lane 2), Δ*ahpC* (lane 4) and Δ*oxyR* (lane 6) strains, which harbored pPROEX-FTN-1133 or pPROEX-OsmC constructions, respectively. As control, the same strains harboring the empty vector were also induced (lanes 1, 3 and 5, respectively). **B.** and **D.** Western blot analysis of the same extracts used in **A.** and **C.** The order of WB lanes was the same presented for Comassie stained gels. Histidine Tag (6×His) Monoclonal Antibody (Novex) was used to detect His-tagged proteins.
(TIF)
