# Supplementary figures and images for: A 14.7 kDa Protein from Francisella tularensis subsp. novicida (Named FTN_1133), Involved in the Response to Oxidative Stress Induced by Organic Peroxides, Is Not Endowed with Thiol-Dependent Peroxidase Activity
Source: PLoS One. 2014 Jun 24;9(6):e99492. doi: 10.1371/journal.pone.0099492 (PMC4069020; doi:10.1371/journal.pone.0099492)

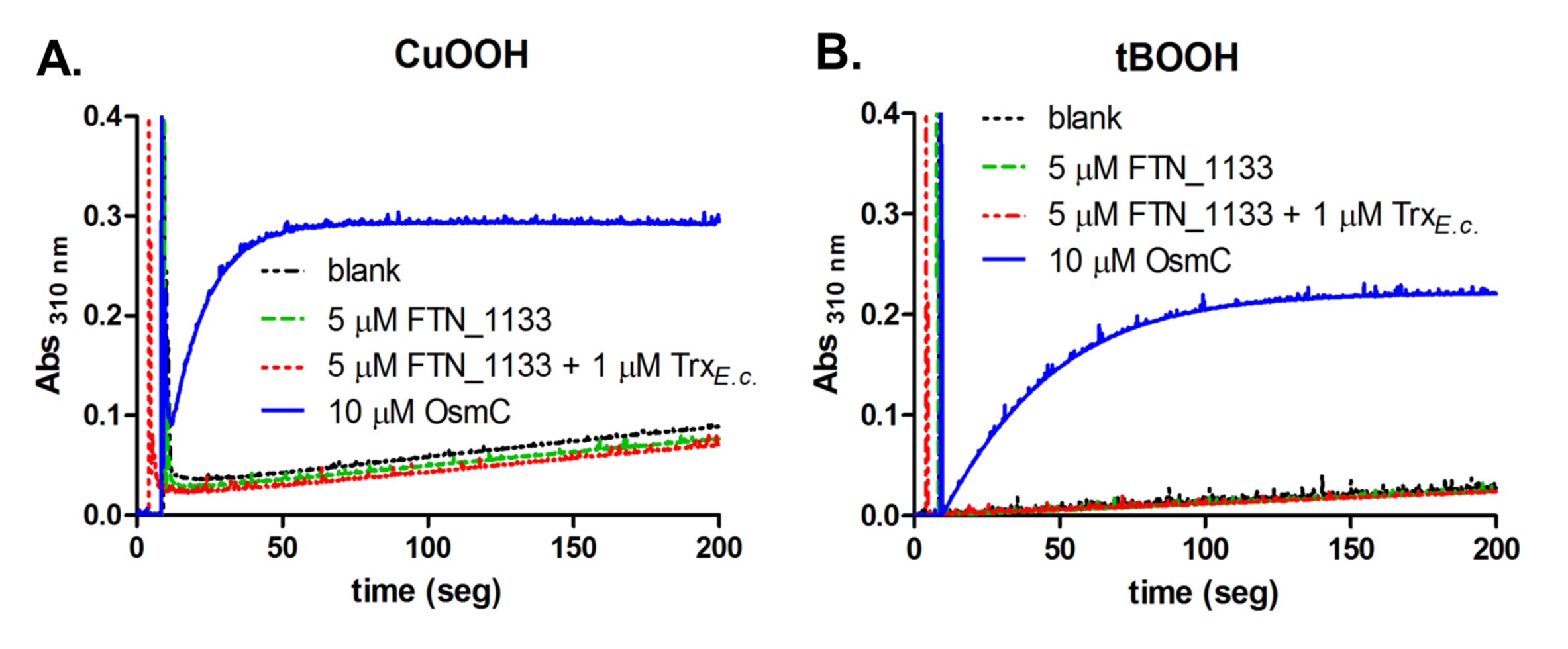

Supplement: Figure S1 — Assay for FTN_1133 thioredoxin-dependent peroxidase activity. Peroxidase activity was followed by generation of oxidized DTT. A. and B. DTT oxidation by CuOOH or tBOOH, respectively. Reactions were carried out in the presence of FTN_1133 (5 µM), sodium phosphate pH 7.4 (100 mM), DTPA (1 mM) and organic hydroperoxide (2 mM), with (red line) or without (green line) addition of 1 µM of recombinant TrxA from E. coli, and started by addition of 10 mM of reduced DTT. As positive control of reaction, the same assay was made using 10 µM of OsmC without addition of TrxA (blue line). The blank reaction (black line) was performed without any enzyme addition. The figure is representative of at least two independent set of experiments. (TIF) [file pone.0099492.s001.tif]

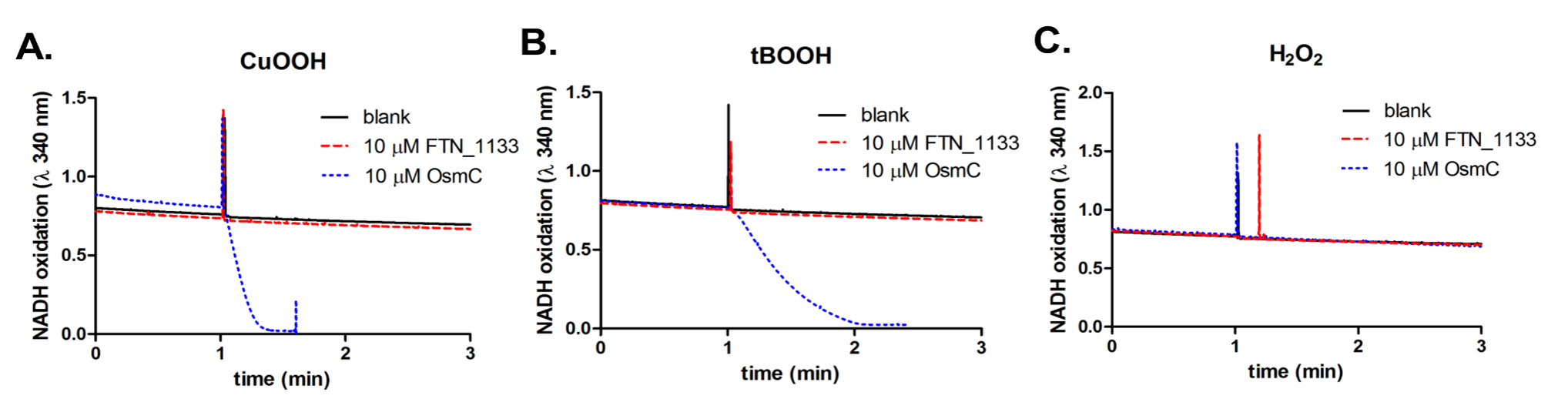

Supplement: Figure S2 — Assay for FTN_1133 lipoyl-dependent peroxidase activity. Lipoamide-Lipoamide dehydrogenase coupled assay was followed decay of absorbance at 340 nm due to NADH oxidation. A., B. and C., represent NADH oxidation in the presence of CuOOH, tBOOH and H2O2 at 37°C, respectively. Reactions were performed with FTN_1133 (10 µM), sodium phosphate pH 7.4 (50 mM), of reduced lipoamide (0.05 mM), DTPA (1 mM), recombinant Lpd (0.005 mM) from Xyllela fastidiosa under gently agitation (red line). After one minute for temperature stabilization, 0.2 mM of NADH was added to reaction that was finally started by addition of 0.2 mM of respective hydroperoxide. As positive control of reaction (blue line), the same assay was carried out using 10 µM of OsmC. The blank reaction (black line) was performed without any enzyme addition. The figure is representative of at least two independent set of experiments. (TIF) [file pone.0099492.s002.tif]

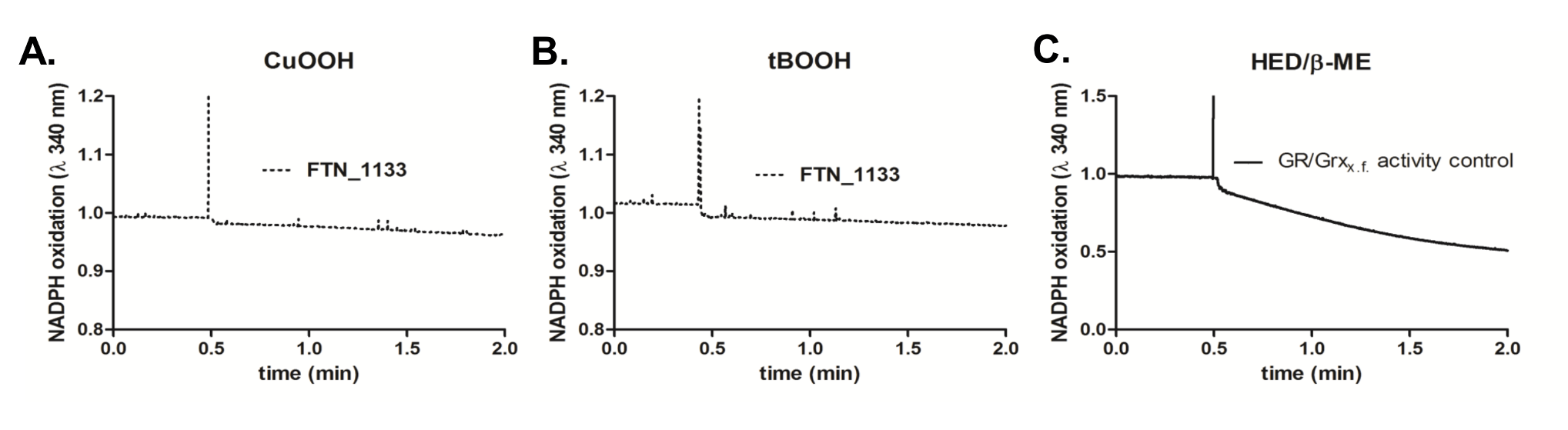

Supplement: Figure S3 — Assay for FTN_1133 Grx/GSH-dependent peroxidase activity. GR/GSH coupled assay was followed by NADPH oxidation. A. and B., NADPH oxidation in the presence of CuOOH and tBOOH at 37°C, respectively. The reaction containing Tris–HCl pH 7.4 (100 mM), yeast GR (6 µg/ml), GrxC (10 µM) from X. fastidiosa, GSH (1 mM), BSA (0.1 mg/ml), DTPA (2 mM) and NADPH (0.2 mM) was initiated by addition of 0.2 mM of CuOOH or tBOOH. C. For GR and GrxC activity control reaction, FTN_1133 and hydroperoxide, were substituted by 0.7 mM of HED that was incubated at 30°C for 3 min for the formation of the mixed disulfide between GSH and HED. The reaction was started by the addition of GrxCx.f. and followed by the decrease in the absorbance at 340 nm due to the oxidation of NADPH [33]. The figure is representative of at least two independent set of experiments. (TIF) [file pone.0099492.s003.tif]

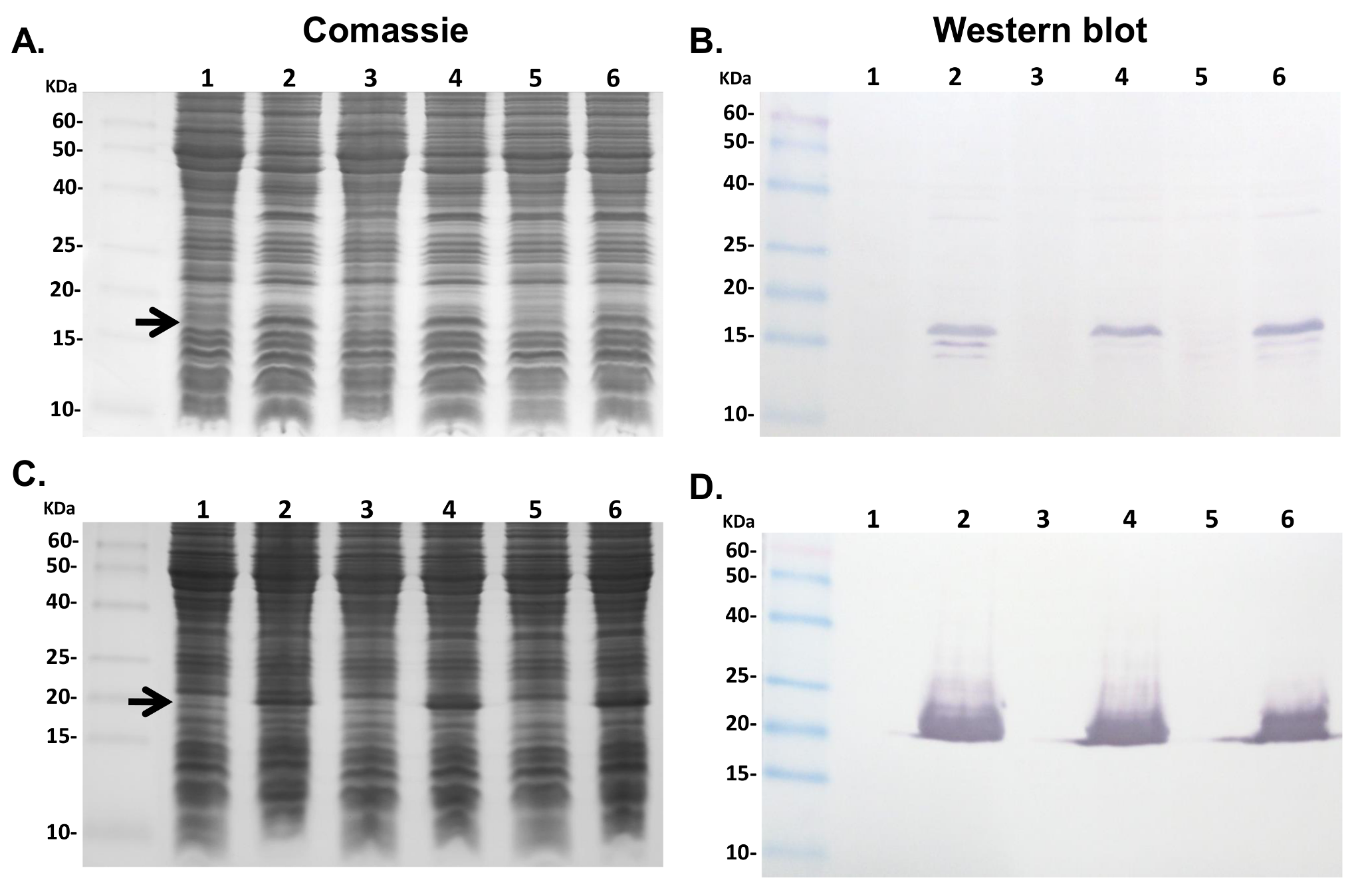

Supplement: Figure S4 — Expression analysis of recombinant FTN_1133 and OsmC proteins in wild type (BW25113), Δ ahpC and Δ oxyR backgrounds. A. and C. Comassie stained gels of total extracts from IPTG induced cultures of wild type BW25113 (lane 2), ΔahpC (lane 4) and ΔoxyR (lane 6) strains, which harbored pPROEX-FTN-1133 or pPROEX-OsmC constructions, respectively. As control, the same strains harboring the empty vector were also induced (lanes 1, 3 and 5, respectively). B. and D. Western blot analysis of the same extracts used in A. and C. The order of WB lanes was the same presented for Comassie stained gels. Histidine Tag (6×His) Monoclonal Antibody (Novex) was used to detect His-tagged proteins. (TIF) [file pone.0099492.s004.tif]
